# The GRP94 Inhibitor PU-WS13 Decreases M2-like Macrophages in Murine TNBC Tumors: A Pharmaco-Imaging Study with ^99m^Tc-Tilmanocept SPECT

**DOI:** 10.3390/cells10123393

**Published:** 2021-12-02

**Authors:** Alexanne Bouchard, Hugo Sikner, Valentin Baverel, Anaïs-Rachel Garnier, Marie Monterrat, Mathieu Moreau, Emeric Limagne, Carmen Garrido, Evelyne Kohli, Bertrand Collin, Pierre-Simon Bellaye

**Affiliations:** 1 Centre George-François Leclerc, Service de Médecine Nucléaire, Plateforme d’imagerie et de Radiothérapie Précliniques, 21000 Dijon, France; abouchard@cgfl.fr (A.B.); hsikner@cgfl.fr (H.S.); anais-garnier@hotmail.fr (A.-R.G.); mmonterrat@cgfl.fr (M.M.); BCollin@cgfl.fr (B.C.); 2 UMR INSERM/uB/AGROSUP 1231, Team 3 HSP-Pathies, Labellisée Ligue National Contre le Cancer and Laboratoire d’Excellence LipSTIC, Université Bourgogne Franche-Comté, 21000 Dijon, France; Valentin_Baverel@etu.u-bourgogne.fr (V.B.); Carmen.Garrido-Fleury@u-bourgogne.fr (C.G.); 3 Institut de Chimie Moléculaire de l’Université de Bourgogne, UMR CNRS/uB 6302, Université de Bourgogne Franche-Comté, 21000 Dijon, France; mathieu.moreau@u-bourgogne.fr; 4 Centre George-François Leclerc, Plateforme de Transfert en Biologie Cancérologique, 21000 Dijon, France; elimagne@cgfl.fr; 5 Centre George-François Leclerc, 21000 Dijon, France; 6 UFR des Sciences de Santé, Université de Bourgogne, 21000 Dijon, France; 7 University Hospital (CHU), 21000 Dijon, France

**Keywords:** GRP94, M2-like macrophages, triple-negative breast cancer, PU-WS13, SPECT imaging, biomarker, CD206, Tilmanocept

## Abstract

Triple-negative breast cancer (TNBC) is the most aggressive subtype of breast cancers and is not eligible for hormone and anti-HER2 therapies. Identifying therapeutic targets and associated biomarkers in TNBC is a clinical challenge to improve patients’ outcome and management. High infiltration of CD206^+^ M2-like macrophages in the tumor microenvironment (TME) indicates poor prognosis and survival in TNBC patients. As we previously showed that membrane expression of GRP94, an endoplasmic reticulum chaperone, was associated with the anti-inflammatory profile of human PBMC-derived M2 macrophages, we hypothesized that intra-tumoral CD206^+^ M2 macrophages expressing GRP94 may represent innovative targets in TNBC for theranostic purposes. We demonstrate in a preclinical model of 4T1 breast tumor-bearing BALB/c mice that (i) CD206-expressing M2-like macrophages in the TME of TNBC can be specifically detected and quantified using in vivo SPECT imaging with ^99m^Tc-Tilmanocept, and (ii) the inhibition of GRP94 with the chemical inhibitor PU-WS13 induces a decrease in CD206-expressing M2-like macrophages in TME. This result correlated with reduced tumor growth and collagen content, as well as an increase in CD8^+^ cells in the TME. ^99m^Tc-Tilmanocept SPECT imaging might represent an innovative non-invasive strategy to quantify CD206^+^ tumor-associated macrophages as a biomarker of anti-GRP94 therapy efficacy and TNBC tumor aggressiveness.

## 1. Introduction

Breast cancer is one of the leading causes of death in women in developed countries. Triple-negative breast cancer (TNBC) represents 15–20% of all breast carcinomas [1]. TNBC is defined by the absence of expression of estrogen receptor (ER), progesterone receptor (PR), and human epidermal growth factor receptor 2 (HER2) [1]. It is the most aggressive subtype of breast cancers coupled with the highest probability of metastasis [2]. Due to the lack of specific targets, hormone therapy and drugs that target HER2 are useless in TNBC [3]. Thus, the identification of therapeutic targets in the tumor microenvironment (TME) of TNBC and associated biomarkers for molecular imaging is a clinical challenge, which may improve patients’ outcome and management. Infiltration of M2-like macrophages in the TME is associated with poor prognosis and survival in TNBC patients [4,5]. Indeed, M2-like macrophages play an essential role in tumor progression by stimulating angiogenesis, tumor cell invasion, and metastasis, and suppressing anti-tumor immunity [6,7,8].

GRP94 (glucose-regulated protein 94), also known as Gp96, endoplasmin, and HSP90b1, is an endoplasmic reticulum (ER) paralog of heat shock protein 90 (HSP90), which is constitutively and ubiquitously expressed. We have previously shown that GRP94 was expressed at the membrane of M2 but not M1 macrophages, which were differentiated in vitro from peripheral blood mononuclear cells (PBMCs) of healthy donors [9]. Its inhibition by a selective purine-based inhibitor, PU-WS13, strongly decreased the presence of iC3b, the inactivated fragment of complement C3b, which has been shown to induce tolerance in macrophages [10]. Additionally, GRP94 has been reported to be associated with gut microbiota immune tolerance and proposed to function as a macrophage tolerance-mediating molecule [11]. Moreover, we showed that GRP94 was co-expressed by about 40% of CD206^+^ M2-like macrophages in murine 4T1 TNBC biopsies [9]. All together, these results lead to the assumption that targeting GRP94 may have an impact on M2-like macrophages in the TME in TNBC. Molecular imaging represents a powerful tool contributing to non-invasive phenotyping of tumors, making it possible to guide targeted therapy. In order to detect M2-like macrophages in solid tumors, Technetium-99m-labeled Tilmanocept (^99m^Tc-Tilmanocept, a diagnostic radiopharmaceutical approved by the U.S. Food and Drug Administration—FDA and European Medicines Agency—EMA) could represent an interesting tool for non-invasive molecular imaging. Indeed, Tilmanocept is a macromolecule consisting of several units of diethylenetriaminepentaacetic acid (DTPA) and mannose, each synthetically linked to a 10 kDa dextran skeleton. DTPA serves as a bifunctional chelating agent for Technetium-99m while mannose acts as the receptor substrate of CD206 (mannose receptor) expressed by M2 macrophages. Currently, it is designed to locate lymph nodes, which may be draining from tumors, and assist surgeons during the removal of lymph nodes. Its clinical routes of administration are subcutaneous/intradermal/intra- or peritumoral. However, several studies recently demonstrated that systemic administration of ^99m^Tc-Tilmanocept allowed the detection of M2-macrophages by single photon emission tomography (SPECT) imaging in several preclinical models of brain injury [12], arthritis [13], and atherosclerosis [14]. In addition, clinical trials are currently underway with systemic administration of ^99m^Tc-Tilmanocept, particularly in oncologic [15,16] and non-oncologic diseases [17].

Given the tumor-promoting role of M2-like macrophages, the aims of our study were to assess if these tumor-associated macrophages (TAM): (i) could be specifically detected in vivo in the TME in a preclinical model of TNBC using ^99m^Tc-Tilmanocept SPECT imaging and (ii) could be modulated by PU-WS13, a cell-permeable 6-amino-purine-based selective inhibitor of GRP94.

## 2. Materials and Methods

### 2.1. Cells Lines and Reagents

The mouse triple-negative 4T1 breast cancer cell line was obtained from the American Type Culture Collection (ATCC^®^ CRL-2539^™^, ATCC, Manassas, VA, USA). PU-WS13 was purchased from Interchim Bioscience, France. The stock solution was prepared in 5% dimethyl sulfoxide (DMSO) (Sigma Aldrich, Louis, MO, USA) and stored at −80 °C until further use.

### 2.2. Cytotoxicity Assay

4T1 cells were cultured in 96-well plates (10,000 cells per well) in 10% fetal bovine serum (FBS) RPMI 1640 medium. Cytotoxicity of PU-WS13 was assessed using the MTS reagent (tetrazolium) according to the recommendations of the supplier (Abcam, France, MTS assay, kit reference 197010). Medium alone or cells treated with 0.1% DMSO were used as controls. MTS reagent was directly added after 24 h of culture and absorbance was read after 1 h at 490 nm. A second cytotoxicity test was carried out to allow the 50% inhibitory concentration (IC_50_) to be calculated at 48 h.

### 2.3. Tilmanocept Radiolabeling

Prior to its radiolabeling, 62.5 µg of Tilmanocept (Lymphoseek^®^, Norgine BV, Amsterdam, The Netherlands) were reconstituted with 200 µL of NaCl 0.9%. Then, a given volume of sodium pertechnetate (^99m^TcO_4_^−^, freshly “milked” from a Tekcis^®^ generator, Curium, Saclay, France) was transferred into a Lobind^®^ (Eppendorf, Hambourg, Germany) microtube with Tilmanocept in order to obtain 2350 ng of Tilmanocept in a final volumic activity of 150 MBq.mL^−1^. The microtube was then placed under agitation at 1000 rpm for 15 min at 25 °C.

The radiochemical purity was checked by radio-ITLC (Instant Thin Layer Chromatography) using a γ-radiochromatograph (AR-2000 Radio-TLC & Imaging Scanner, Bioscan, Washington, DC, USA). Briefly, 1μL of solution was deposited on cellulose chromatographic paper (Whatman Grade 1, 3MM, 31ET Chr, Merck, France) and eluted with acetone to separate the ^99m^TcO_4_^−^ (“free” Tc-99m) from ^99m^Tc linked to Tilmanocept. On the chromatographic strip, the ^99m^Tc-Tilmanocept remains at the deposition point while the “free” Tc-99m migrates to the solvent front. The radiochromatograph obtained allows determination of the radiochemical purity by integration of the different peaks. Results were obtained using the WinScan^®^ software (Bioscan, Washington, DC, USA).

### 2.4. In Vivo Experiments

#### 2.4.1. Triple-Negative Breast Cancer Model

All animal studies were performed in compliance with preclinical ethical guidelines and approved by the local ethical committee (C2EA Grand Campus, project #13445, Dijon, France). A syngeneic orthotopic murine model of TNBC was used. A total of 5 × 10^4^ 4T1 cells were administered using a 27G needle (total volume of 50 µL in RPMI 1640 medium without fetal bovine serum FBS) into the mammary fat pad of immunocompetent 7-week-old female BALB/c mice (Charles River, Ecully, France). All the administrations of cells were performed under anesthesia by isoflurane 2%.

Prior to in vivo implantation, 4T1 cells were cultured in RPMI medium supplemented with 10% FBS at 37 °C in a CO_2_ incubator. All animals were kept under pathogen-free conditions at 22 °C, 33% relative humidity (RH), with a controlled 12 h day-night cycle, and had free access to standard diet and mineral water. Tumor volume (TV) was monitored daily with a caliper and calculated as TV = (x × y^2^)/2, where x is the largest diameter and y is the corresponding orthogonal diameter.

#### 2.4.2. In Vivo Imaging

The 4T1 tumor-bearing mice (*n* = 14) were divided into two groups 21 days after implantation. The first group (*n* = 8) received a volume activity ratio of 15 MBq/100 µL per mouse corresponding to a final concentration of Tilmanocept of 2350 ng/mL, by IV injection of the radiolabeled compound in the tail vein under anesthesia (isoflurane 2%). The second group (blocking group, *n* = 6) received an excess of 20-fold of non-radioactive (cold) Tilmanocept to visualize the competition of binding between radioactive and non-radioactive probes. This group received a volume activity ratio of 15 MBq/100 µL per mouse corresponding to a final concentration in Tilmanocept of 47,000 ng/mL. Simultaneous SPECT/CT images (NanoSPECT/CT^®^, Mediso, Budapest, Hungary) were acquired at 1 h post injection. After 24 h, mice were euthanized and tumors and blood were collected for γ-counting (Wizard^®^ 1480, PerkinElmer, Villebon-sur-Yvette, France). Tumors were collected in formalin 10% for 24 h and then transferred in ethanol 70% for histological analysis.

#### 2.4.3. Pharmacological Study

At day 11, mice (*n* = 17) were randomized into two groups receiving either the GRP94 inhibitor PU-WS13 (daily IP, 0.3 mg/injection, *n* = 9) or vehicle (NaCl, 5% DMSO, *n* = 8). Treatments were performed from day 11 until day 22. Then, ^99m^Tc-Tilmanocept SPECT/CT imaging was performed as described above.

After 24 h, mice were euthanized and tumors and blood were collected for γ-counting (Wizard^®^ 1480, PerkinElmer, Villebon-sur-Yvette, France). Tumors were collected in formalin 10% for 24 h and then transferred in ethanol 70% for histological analysis.

### 2.5. PU-WS13 Dosage in Tumor

Mice bearing 4T1 tumors were treated with PU-WS13 (15 mg/kg) every day from day 11 post tumor implantation. Mice were sacrificed 24 h after one dose (day 12, *n* = 3), five doses (day 15, *n* = 3), or 11 doses (day 22, *n* = 3) of PU-WS13. Tumors were collected and frozen at −80 °C. After thawing, tumors were homogenized in PBS before being crushed. PU-WS13 was extracted in acetonitrile, and the organic layer separated and dried under vacuum. Samples were reconstituted in mobile phase. Concentrations of PU-WS13 in tumors were determined by high-performance LC-MS/MS. PU-H71 was added as the internal standard. Compound analysis was performed on a 6460 LC-MS/MS system (Agilent, Les Ulis, France).

### 2.6. Immunofluorescence

Mouse tumor 4T1 and human TNBC biopsy sections were successively immersed in xylene (3×, 5 min), ethanol 100% (2×, 5 min), ethanol 90% (1×, 5 min), and 70% (1×, 5 min) before being rehydrated in a PBS-Tween 0.1% (PBS-T) buffer (2× 3 min). Antigen unmasking was achieved by heating at 95 °C in a 10 mM citrate buffer for 30 min and slides were cooled for 30 min at room temperature (RT). Saturation was performed by 1 h incubation with a PBS-T, 8% BSA solution. The tissues were then stained with a rabbit polyclonal antibody directed against CD206 (1/100; LS-B9805-200, Clinisciences, Nanterre, France), GRP94 antibody (1/100; LS B3418, Clinisciences, Nanterre, France), or against CD8 (1/200; NBP2-29475, Novusbio, Noyal Châtillon sur Seiche, France) overnight at 4 °C. The tissues were then washed 3 times with PBS-T and incubated for 1 h at RT with an anti-rabbit Alexa 568 (Abcam, ab175470, Cambridge, UK). Slides were then washed 3 times and mounted with a cover slide using mounting medium with DAPI (Thermo Fisher Scientific, Waltham, MA, USA) for nuclear staining. Finally, slides were observed using a fluorescence microscope (Zeiss, Oberkochen, Germany) and analyzed with ZEN^®^ software (Zeiss, Oberkochen, Germany) and quantified by the open-source program (ImageJ/Fiji software, NIH, Bethesda, MD, USA). Briefly, each color channel (DAPI, blue and CD8, green) was separated. The area represented by CD206 or CD8 staining was quantified ImageJ software and expressed as the percentage of total tumor section area.

### 2.7. Collagen Quantification

The amount of collagen in paraffin-embedded tissue sections was quantified by histochemical staining with Picrosirius red as previously described [18]. Briefly, 10 random fields for each tumor were digitized under polarized transmission illumination. The collagen amount was measured using 20 circles/section, placed randomly throughout the tumor; the circles had a constant diameter of 64 µm. The collagen content within the circles was expressed as the percentage of emission (ImageJ software). Collagen width was measured using the ImageJ software at 10 random locations around each tumor.

### 2.8. Western Blot

Tumors were lysed in RIPA lysis buffer. Samples (20 µg of protein) were prepared in classical loading buffer and loaded on 12% SDS-PAGE gel before being transferred to the PVDF membrane. Immunoblot was performed overnight at 4 °C with the anti-CD8 antibody (1:1000, NBP2-29475, Novusbio, Noyal Châtillon sur Seiche, France). β-actin (ab8227, Abcam, Cambridge, UK) antibody was used as a loading control. A secondary anti-rabbit antibody, conjugated with horseradish peroxidase (HRP), was used (1:5000, ab205722, Abcam, Cambridge, UK).

### 2.9. SPECT/CT Image Analysis

All SPECT/CT fusion images were obtained using the VivoQuant^TM^ software (Invicro, Needham, MA, USA). Each image was visually interpreted and 3D regions of interest (3D ROI) corresponding to the tumors were manually drawn to determine their radioactivity content. Injected doses per animal were measured at the time of injection in MBq. Tumor radioactivity content was expressed in MBq, converted to percentage of injected dose per gram of tissue (%ID/g). All images were decay corrected for quantification.

### 2.10. Autoradiography

After deparaffination (Xylene) and antigen unmasking (30 min in citrate buffer pH 6) sections from human TNBC biopsies were saturated (BSA 8%) and incubated for 1 h with ^99m^Tc-Tilmanocept (1 MBq, 235 ng/slide) or with ^99m^Tc-Tilmanocept (1 MBq, 235 ng/slide) and an excess (100 fold) of unlabeled Tilmanocept (blocking). After 4 washes with cold DPBS, slides were exposed to phosphor imaging plates (Fuji imaging plates, Fujifilm, Tokyo, Japan). After 2–3 h of exposure time, the imaging plates were scanned and the autoradiograms were obtained with a phosphor imaging system (GE, Amersham, Molecular Dynamics, Chicago, IL, USA). Images were analyzed for count densities with imageJ software. Data were used to calculate the autoradiographic signal intensity in TNBC biopsies.

### 2.11. Statistical Analysis

For all experiments, statistical analyses were performed by non-parametric Mann–Whitney t-tests using GraphPad prism 8.0 (GraphPad software, San Diego, CA, USA). All results are represented as the median with an interquartile range. The level of significance was set at *p* < 0.05.

## 3. Results

### 3.1. GRP94 Is Co-Expressed by CD206^+^ M2-like Macrophages in Murine 4T1 and Human TNBC Infiltrated with CD206^+^ Cells

**Figure 1:**
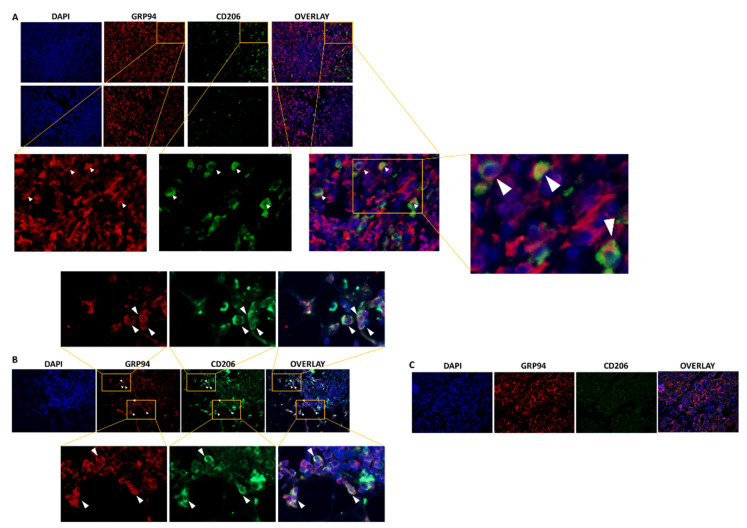
GRP94 and CD206 are co-expressed in murine and some human TNBC tumors. (**A**). Representative immunostaining of GRP94 (red) and CD206 (green) in 4T1 tumor-bearing mice (*n* = 8). (**B**). Representative immunostaining of GRP94 (red) and CD206 (green) in a biopsy from a human TNBC tumor infiltrated with CD206^+^ M2-like macrophages (*n* = 3). (**C**). Representative immunostaining of GRP94 (red) and CD206 (green) in a biopsy from a human TNBC tumor non infiltrated with CD206^+^ M2-like macrophages (*n* = 3).

We first used immunofluorescence to confirm previous preliminary results showing the presence of CD206^+^ M2-like macrophages in 4T1 biopsies, among which, 44% highly expressed GRP94 [9]. As shown in Figure 1A, CD206^+^ macrophages were present in the 4T1 biopsies analyzed and 35.8% ± 6.40% co-expressed GRP94 in coherence with our previous results. Moreover, we analyzed human TNBC biopsies and showed that some tumors were infiltrated with CD206^+^ M2-like macrophages co-expressing GRP94 (Figure 1B) whereas other tumors were not (Figure 1C). Of importance, GRP94 was also overexpressed by some 4T1 cells.

### 3.2. SPECT/CT Imaging with ^99m^Tc-Tilmanocept Allows Specific Detection of CD206^+^ Macrophages in Murine TNBC In Vivo

Based on these results, we thought to detect CD206^+^ cells in vivo by SPECT/CT imaging using the CD206 targeting agent ^99m^Tc-Tilmanocept.

**Figure 2:**
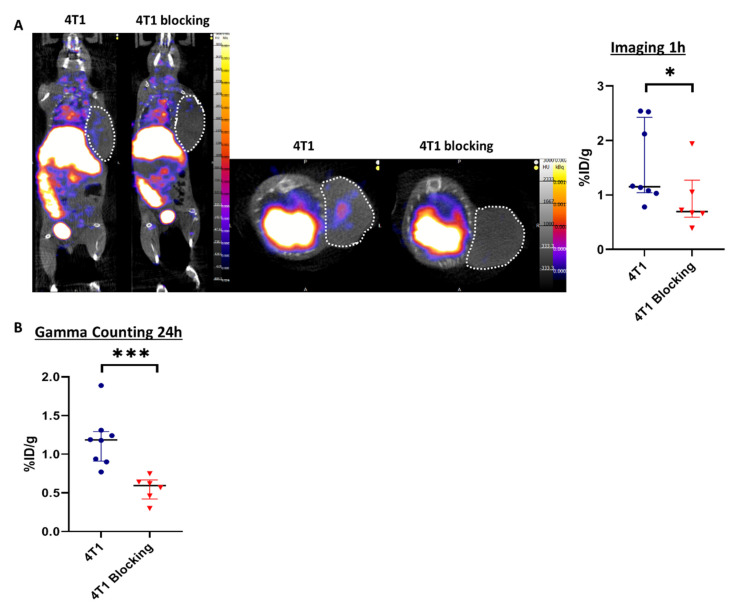
SPECT/CT imaging with ^99m^Tc-Tilmanocept can specifically detect CD206^+^ cells in vivo. (**A**) Representative SPECT/CT images of 4T1 tumor-bearing mice at 1 h post-injection of 15 MBq/100 µL of ^99m^Tc-Tilmanocept (4T1, *n* = 8) or 15 MBq/100 µL of ^99m^Tc-Tilmanocept with a 20× excess of cold Tilmanocept (4T1 blocking, *n* = 6). Results are presented as the median with the interquartile range. The scatter dot plot represents the percentage of the injected dose of ^99m^Tc-Tilmanocept per gram of tumor (%ID/g) for both groups (* *p* = 0.0293). (**B**) The scatter dot plot represents the percentage of injected dose of ^99m^Tc-Tilmanocept per gram of tumor of 4T1 tumor-bearing mice at 24 h post-injection of 15 MBq/100 µL of ^99m^Tc-Tilmanocept (4T1, *n* = 8) or 15 MBq/100 µL of ^99m^Tc-Tilmanocept with a 20x excess of cold Tilmanocept (4T1 blocking, *n* = 6) measured by gamma counting (*** *p* = 0.0007).

Tilmanocept was radiolabeled with ^99m^Tc and the radiochemical purity of the radiolabeling was checked by radio-ITLC prior to in vivo administration. The results showed a purity above 90%, which was suitable for in vivo experiments (Figure S1), according to the manufacturer’s summary of product characteristics. Two groups of mice were injected with ^99m^Tc-Tilmanocept, one of them also receiving a 20× higher dose of cold Tilmanocept (blocking group) to confirm the specificity of labeling. At 1h post-injection, the ^99m^Tc-Tilmanocept tumor uptake was significantly increased in 4T1 tumors compared to mice receiving a concomitant excess of unlabeled Tilmanocept (1.55 ± 0.30 %ID/g vs. 0.90 ± 0.22 %ID/g, *p* = 0.0293), thus demonstrating the specificity of this probe for 4T1 tumors (Figure 2A). Ex vivo radioactivity quantification of tumors 24 h post-^99m^Tc-Tilmanocept injection confirmed the increase in ^99m^Tc-Tilmanocept uptake compared with the blocking group (1.18 ± 0.12 %ID/g vs. 0.56 ± 0.10 %ID/g, *p* = 0.0007) (Figure 2B). These results were also confirmed ex vivo by autoradiography on human TNBC biopsies in which ^99m^Tc-Tilmanocept autoradiogaphic signal was significantly increased compared to biopsies receiving a concomitant excess of unlabeled Tilmanocept (2.079 ± 0.15 AU vs. 1.354 ± 0.26 AU, *p* = 0.0286) (Figure S2).

### 3.3. GRP94 Inhibition by the Chemical Inhibitor PU-WS13 Decreases ^99m^Tc-Tilmanocept Tumor Uptake In Vivo

**Figure 3:**
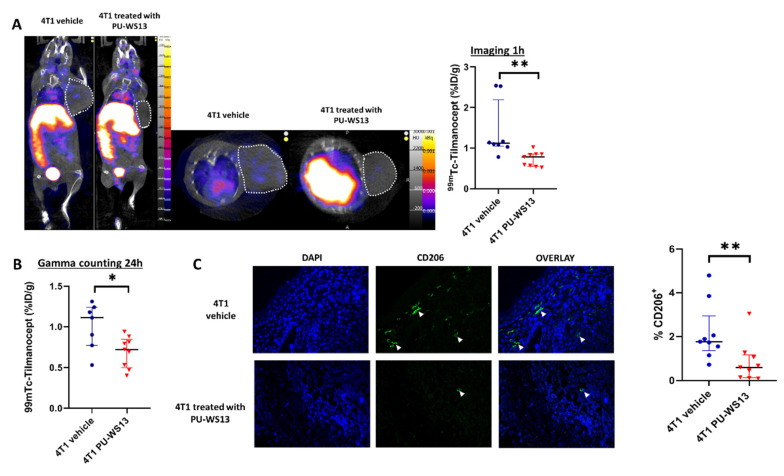
GRP94 inhibition by PU-WS13 induces a decrease in ^99m^Tc-Tilmanocept tumor uptake in vivo. (**A**). Representative SPECT/CT images of 4T1 tumor-bearing mice receiving vehicle (*n* = 8) or PU-WS13 (*n* = 9) from D11 up to D22 at 1 h post-injection of 15 MBq/100 µL of ^99m^Tc-Tilmanocept. Tumors are highlighted with circles. The scatter dot plot represents the percentage of the injected dose of ^99m^Tc-Tilmanocept per gram of tumor (%ID/g) for both groups. Results are presented as the median with the interquartile range, ** *p* = 0.0011. (**B**). The scatter dot plot represents the percentage of injected dose of ^99m^Tc-Tilmanocept per gram of tumor of 4T1 tumor-bearing mice receiving vehicle (*n* = 8) or PU-WS13 (*n* = 9) from D11 up to D22 at 1 h post-injection of 15 MBq/100 µL of ^99m^Tc-Tilmanocept measured by gamma counting. Results are presented as the median with the interquartile range. * *p* = 0.0328. (**C**). Representative images (vehicle *n* = 9, PU-WS13 *n* = 9) and quantification of CD206 staining in 4T1 tumors from mice treated or not with PU-WS13 from D11 up to D22. Results are presented as the median with the interquartile range. ** *p* = 0.0056.

4T1 tumor-bearing mice were injected with the GRP94 inhibitor PU-WS13 or vehicle only from day 11 to day 22 after tumor implantation. They underwent SPECT/CT imaging at 1 h post-injection with ^99m^Tc-Tilmanocept. At 1 h post-injection, the tumor uptake of ^99m^Tc-Tilmanocept was significantly higher in the vehicle group compared with mice receiving PU-WS13 (1.42 ± 0.25 %ID/g vs. 0.73 ± 0.06 %ID/g, *p* = 0.0011) (Figure 3A). Similar results were found by ex vivo gamma counting at 24 h post-injection (1.01 ± 0.11 %ID/g vs. 0.70 ± 0.06 %ID/g, *p* = 0.0328) (Figure 3B). Further, immunostaining of tumors from 4T1 tumor-bearing mice treated with PU-WS13 showed a significant decrease in the percentage of CD206^+^ cells compared with untreated mice in correlation with in vivo imaging findings (0.82 ± 0.31 %ID/g vs. 2.16 ± 0.44 %ID/g of nucleated cells in the tumor, *p* = 0.0056) (Figure 3C).

### 3.4. GRP94 Inhibition by PU-WS13 Limits Tumor Growth and Collagen Content and Increases CD8^+^ Cells in the TME

**Figure 4:**
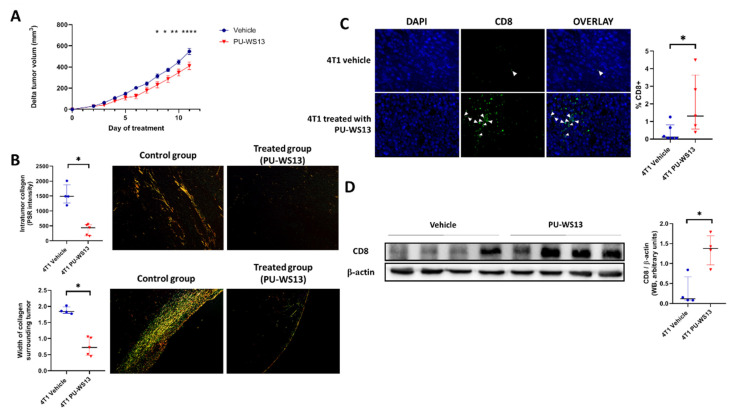
GRP94 inhibition by PU-WS13 limits tumor growth and collagen content and increases CD8^+^ cells in the TME. (**A**). 4T1 tumor growth (mm^3^) measured daily during PU-WS13 (*n* = 15) or vehicle (*n* = 20) treatment from D11 up to D22 post implantation. Results are presented as the median with the interquartile range. *p* = 0.0436 and 0.0325, ** *p* = 0.0061, **** *p* < 0.0001, *n* = 20 for vehicle group and n = 15 for PU-WS13 group. (**B**). PicoSirius red staining of 4T1 tumors treated or not with PU-WS13 showing the collagen intensity within the tumor and the width (mm) of collagen surrounding the tumor. Results are presented as the median with the interquartile range. * *p* = 0.0159, *n* = 6 per group. (**C**). Representative images (*n* = 6 vehicle and *n* = 6 PU-WS13) and quantification of CD8 staining of 4T1 tumors treated or not by PU-WS13. Results are presented as the median with the interquartile range. * *p* = 0.0303. (**D**). Western blot analysis of CD8^+^ in 4T1 tumors treated or not by PU-WS13 (*n* = 4 vehicle and *n* = 4 PU-WS13), * *p* = 0.0286.

Tumor volume of 4T1 tumor-bearing mice treated with PU-WS13 or vehicle was measured daily from the first day of treatment (i.e., day 11 post-implantation) to the end (i.e., day 22 post-implantation). PU-WS13 induced a significant reduction in tumor growth from day 8 post-treatment (*p* = 0.0402) which increased until the end of the treatment compared to untreated mice (*p* < 0.0001 at day 11 post-treatment) (Figure 4A). This reduction in tumor size was associated with a significant decrease in the intratumoral collagen content (374 ± 83.0 PSR intensity vs. 1547 ± 171 PSR intensity, *p* = 0.0159) measured by Picrosirius red-specific staining along with a significant reduction of the width of collagen surrounding 4T1 tumors (0.75 ± 0.13 mm vs. 1.90 ± 0.05 mm, *p* = 0.0159) (Figure 4B).

We then analyzed whether the decrease in intratumoral collagen was associated with a higher infiltration of CD8^+^ cells in the tumor by immunochemistry. Immunostaining showed a significant increase in CD8^+^ cells in the TME for mice treated with PU-WS13 compared to untreated mice (1.95% ± 0.76% vs. 0.37% ± 0.20% of nucleated cells in the tumor, *p* = 0.0303) (Figure 4C). This result was in accordance with a significant increase in CD8 expression measured in tumors by Western blot (1.35 ± 0.20 AU vs. 0.30 ± 0.18 AU *p* = 0.0286) (Figure 4D).

## 4. Discussion

Immunosuppression is a key component of the TME and is a well-known immune evasion hallmark of cancer. Among TME cells, M2-like macrophages are recognized as playing a major role in tumor progression by stimulating angiogenesis, tumor cell invasion, and metastasis, and suppressing anti-tumor immunity mainly through the secretion of cytokines, such as TGF-β [7,8]. The increasing number of studies showing the impact of macrophages on tumor development as well as their potential as prognostic markers of tumor aggressiveness and progression highlight the relevance of macrophages as a promising therapeutic and imaging target in cancer therapy and monitoring [19,20]. In TNBC, the presence of infiltrating M2-like macrophages within tumors is correlated with poor prognosis and survival [4,5]. Moreover, as they can directly and indirectly modulate the expression of programmed cell death 1 (PD-1) and PD-ligand (L) 1 in the tumor environment, targeting of M2-like TAM has been proposed to represent an effective approach in TNBC to modulate the activity of immune checkpoint inhibitors [21].

In this study, we first confirmed previous ex vivo preliminary results, which showed the presence of CD206^+^ M2-like macrophages in the TME of murine 4T1 tumors, a high percentage of them expressing GRP94 [9]. Moreover, we showed the presence of CD206^+^ M2-like macrophages co-expressing GRP94 in human TNBC tumors. Finally, we could detect the presence of CD206^+^ macrophages in 4T1 tumors using an innovative molecular imaging technique based on the uptake of ^99m^Tc-Tilmanocept. Indeed, we were able to demonstrate that SPECT/CT was a technique sensitive and quantitative enough to detect and measure a low but significant increase in CD206^+^ cells in tumors. The specificity of this uptake was demonstrated in vivo in our murine model through blocking experiments (addition of a 20-fold excess of non-radiolabeled Tilmanocept), which induced a signal decrease of about 40%. These results were confirmed ex vivo by autoradiography on human TNBC biopsies in which ^99m^Tc-Tilmanocept autoradiogaphic signal was significantly increased compared to biopsies receiving a concomitant excess of non-radiolabeled Tilmanocept. The relatively low uptake values observed in our study are in accordance with our immunofluorescence results, which show that CD206^+^ macrophages represent less than 4% of all nucleated cells in the 4T1 tumor and with published results reporting 6% of M2-like macrophages among CD45^+^ cells, i.e., about 3% of all tumor cells [22].

On the basis of these results, we hypothesized that GRP94 overexpression by M2-like macrophages could be associated with a tolerogenic phenotype as already shown for intestinal macrophages by Schreiter et al. [11] and therefore could represent an interesting target to impact M2-like macrophages in cancer. We used PU-WS13, a cell-permeable 6-amino-purine-based selective inhibitor of GRP94, to treat 4T1 tumor-bearing mice and showed, using SPECT/CT with ^99m^Tc-Tilmanocept, a significant decrease in CD206^+^ M2-like macrophages after 11 days of treatment. This result was confirmed by staining CD206^+^ macrophages in tumor biopsies using immunofluorescence. Thus, as hypothesized, GRP94 targeting has an impact on M2-like macrophages in our 4T1 model. Moreover, we show that it is possible to non-invasively follow these macrophages in real time by SPECT/CT with ^99m^Tc-Tilmanocept.

The significant decrease in CD206^+^ M2-like macrophages induced by PU-WS13 was associated with reduced tumor growth as well as a lower collagen content, both inside and around the tumors. Our data are supported by the previous work of Acerbi et al., who established a link between collagen and TAM through the highest level of TGF-β signaling in a human breast cancer model [23]. TAM have been demonstrated to play a role in collagen deposition and to support the formation of a dense extracellular matrix (ECM) [24,25]. These findings suggest that the reduction of collagen observed in our model may be an indirect effect of GRP94 inhibition, which decreases M2-like macrophages in tumors, leading to a decrease in TGF-β in the tumor microenvironment. Nevertheless, a direct effect of GRP94 inhibition on collagen deposition may not be excluded as GRP94 has been implicated in procollagen maturation events along with other ER chaperons, HSP47 and GRP78, especially in stress conditions [26]. Indeed, GRP94 has been shown to form heterocomplexes with GRP78 and HSP47 to promote the maturation of newly synthesized collagen and delays in collagen maturation have been reported in the condition of GRP78/GRP94 inactivation [26,27]. Further investigation is needed to enlighten the exact role of GRP94 inhibition in collagen maturation and secretion.

In addition, the structure of the ECM controls T cell migration within tumors and their ability to contact malignant cells [28,29]. In accordance with our results showing a low number of CD8^+^ cells within untreated 4T1 tumors, Kuczek et al. observed a reduction in the number of infiltrating CD8^+^ T cells in mammary TNBC with high collagen density [30]. Furthermore, Peranzoni et al. demonstrated that TAM are the key determinant of the establishment of a T cell-excluded tumor phenotype in lung cancer. TAM can reduce the motility of CD8 T cells, limiting their entry into the tumor. Moreover, a depletion of TAM with a specific inhibitor restored CD8 T cell migration and infiltration into the tumor [31]. Therefore, the increase in the number of CD8^+^ cells upon PU-WS13 treatment in our preclinical model, which is associated with reduced tumor growth, might be due to the decrease in tumoral collagen content, facilitating the infiltration of CD8 T cells or/and to the decrease in M2-like macrophages and its consequences on the mobility of CD8^+^ T cells and infiltration into the tumor [31].

GRP94 chaperones several oncogenic and immunomodulatory proteins (reviewed in Duan et al. [32]), so the decrease in M2-like macrophages observed with PU-WS13 may be the consequence of a direct effect on CD206^+^ macrophages or an indirect effect, especially as GRP94 is also expressed by 4T1 cells [9]. Among oncogenic proteins that are chaperoned by GRP94, lipoprotein receptor-related protein 6 (LRP6), an indispensable co-receptor for WNT, insulin-like growth factor (IGF), and integrins are of particular importance in TNBC [33,34,35]. Besides its role in chaperoning oncogenic proteins, GRP94 has been shown to protect EGFR from degradation [36], a receptor that is frequently overexpressed in TNBC [37]. Moreover, Yan et al. reported complete tumor regression associated with a decrease in EGFR expression and an increase in tumor cell apoptosis in a murine xenograft model of TNBC using high dosages of PU-WS13 [38]. In comparison with our study, Yan et al. [38] used higher doses of PU-WS13 and observed a PU-WS13 concentration in tumors in the µM range while with our protocol, the PU-WS13 concentration in tumors was in the nM range (Figure S3). This discrepancy may be explained by higher doses and longer treatment schedules than in our model. In any case, the amount of PU-WS13 found in our preclinical model is likely too low to induce apoptosis of 4T1 cancer cells with regard to the IC_50_ of PU-WS13 that we found in vitro on 4T1 cells (12.63 µM, Figure S3). Therefore, the effect of PU-WS13 at the dose used in our study (15 mg/kg) may be more related to an immunomodulatory effect rather than to a direct pro-apoptotic effect on tumor cells. We previously showed that PU-WS13 decreased the inactivated fragment of complement C3b, iC3b, at the surface of M2 macrophages [9]. iC3b has been shown to induce tolerance in macrophages [10] and dendritic cells [39], thus its decrease may slow down immunosuppression. Another effect could result from the role of GRP94 in the activation of TGF-β, another major immunosuppressive molecule, as GRP94 chaperones glycoprotein-A repetition predominant (GARP, [40]) and integrins αvβ6 and αvβ8 [41], which are involved in the release of biologically active TGF-β. Although GARP is not expressed by M2 macrophages [9], it can be widely present in tumors, both on tumor cells and on the cells of the TME [42], thus promoting oncogenesis by positively regulating TGF-β in the TME [43]. TGF-β influences the polarization of macrophages toward an M2-like phenotype [7,8] and has many critical roles in numerous aspects of biological processes [43]. In cancer, TGF-β induces extracellular matrix deposition [44,45] that may explain the decrease of intratumoral collagen after treatment with PU-WS13.

TAM have been reported to modulate PD-1 and PD-L1 in the tumor environment and reducing TAM has been proposed as a promising strategy to enhance anti-checkpoint inhibitor efficacy [21,46]. Moreover, the clinical response to anti-PD-1 immunotherapies is associated with the presence of CD8^+^ T cells in tumor cells before the treatment [47,48]. If the accumulation of CD8^+^ T cells is not sufficient, patients harboring this tumor phenotype do not respond to anti-PD-1 antibodies. Our study demonstrates a significant decrease in tumor volume in a syngeneic model of TNBC with low doses of PU-WS13 associated with an increase in CD8 cells in the TME. To markedly decrease tumor growth in TNBC and avoid potential toxicity associated with high dosages in humans, it could be interesting to use the GRP94 inhibitor in combination with/or before an immune checkpoint inhibitor, such as anti-PD-L1 antibodies, which are already used in clinics and in several clinical trials in TNBC [49]. By promoting tumor repopulation by CD8^+^ T cells, a GRP94 inhibitor may improve the efficacy of immune checkpoint inhibitors.

## 5. Conclusions

Since myeloid cells, such as M2-like macrophages, represent a major component of the TME, molecular imaging modalities with exquisite sensitivity, such as SPECT, should be used to non-invasively track and monitor TAM dynamics, especially in the field of immunotherapies. In this context, ^99m^Tc-Tilmanocept SPECT made it possible to perform molecular imaging of TAM in a TNBC preclinical model, thus allowing its use for personalized care and therapeutic follow-up in TNBC as ^99m^Tc-Tilmanocept is already approved in humans. Moreover, our results with the GRP94-specific inhibitor PU-WS13 highlight a link between GRP94 and the number of CD206^+^ M2-like macrophages, the intratumoral content of collagen, the number of infiltrating CD8^+^ cells, and the development of the tumor. The immediate downstream mechanisms of GRP94 in regulating the infiltration of M2-like macrophages as well as those impacting tumor growth remain to be demonstrated. The decrease in tumor growth may be the only consequence of the decrease in M2-like macrophages, but effects on other immunosuppressive cells cannot be excluded as GRP94 inhibition impacts iC3b and GARP, which are major immunosuppressive effectors.

## Data Availability

All data will be available on request and after examination and will require the signature of a materials transfer agreement (MTA) between parties.

## Supplementary Materials

The following are available online at https://www.mdpi.com/article/10.3390/cells10123393/s1, Figure S1: ITLC radiochromatograms of ^99m^Tc-Tilmanocept (left) and free Tc-99m (right); Figure S2: ^99m^Tc-Tilmanocept autoradiography on human TNBC biopsies; Figure S3: Tumor growth inhibition by PU-WS13 is independent of tumor cell death.
