# Supplementary figures and images for: The GRP94 Inhibitor PU-WS13 Decreases M2-like Macrophages in Murine TNBC Tumors: A Pharmaco-Imaging Study with 99mTc-Tilmanocept SPECT
Source: Cells. 2021 Dec 2;10(12):3393. doi: 10.3390/cells10123393 (PMC8699502; doi:10.3390/cells10123393)

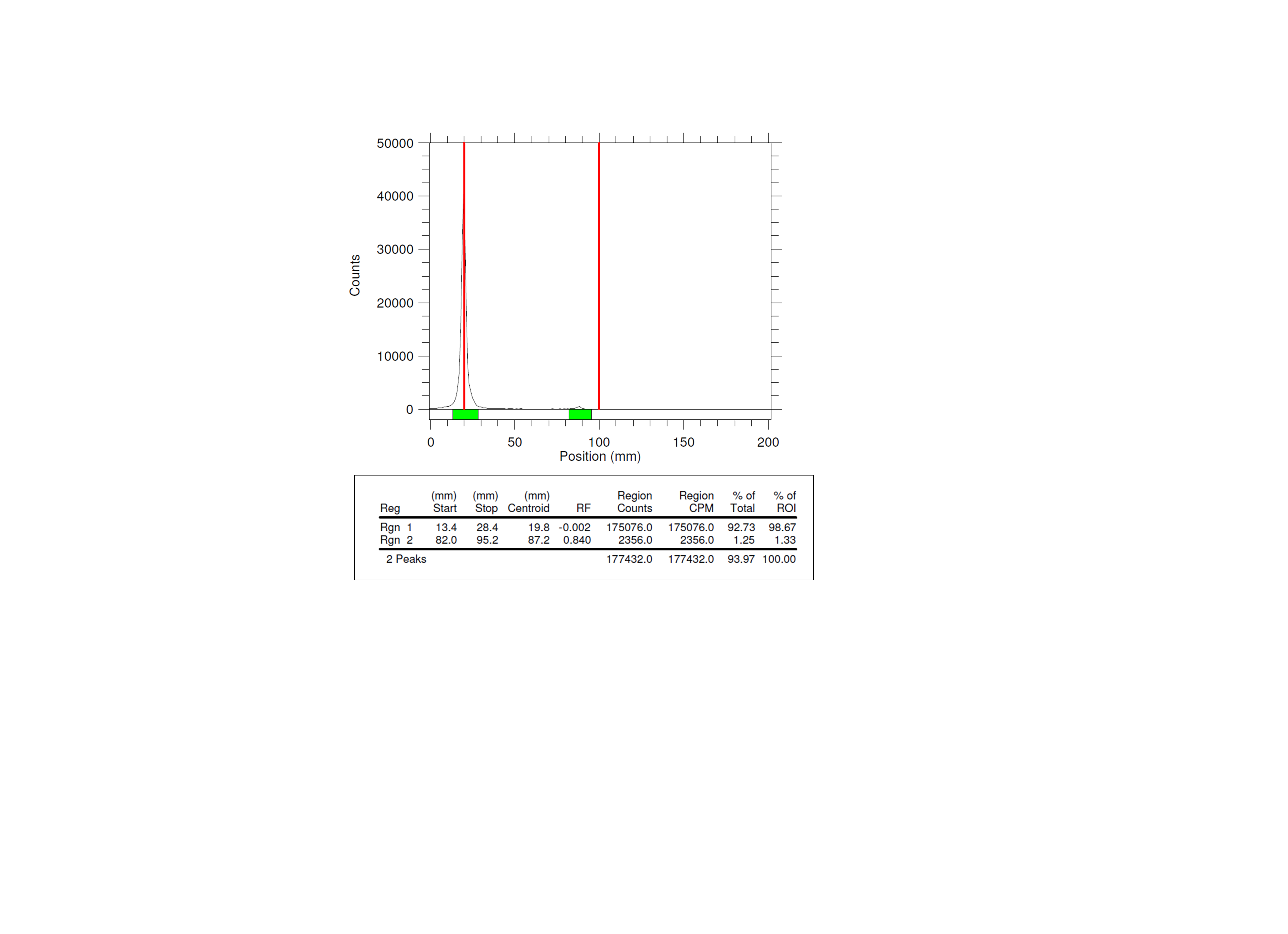

Supplement: Supplementary file 1 [file cells-10-03393-s001.zip › Figure S1.TIF]

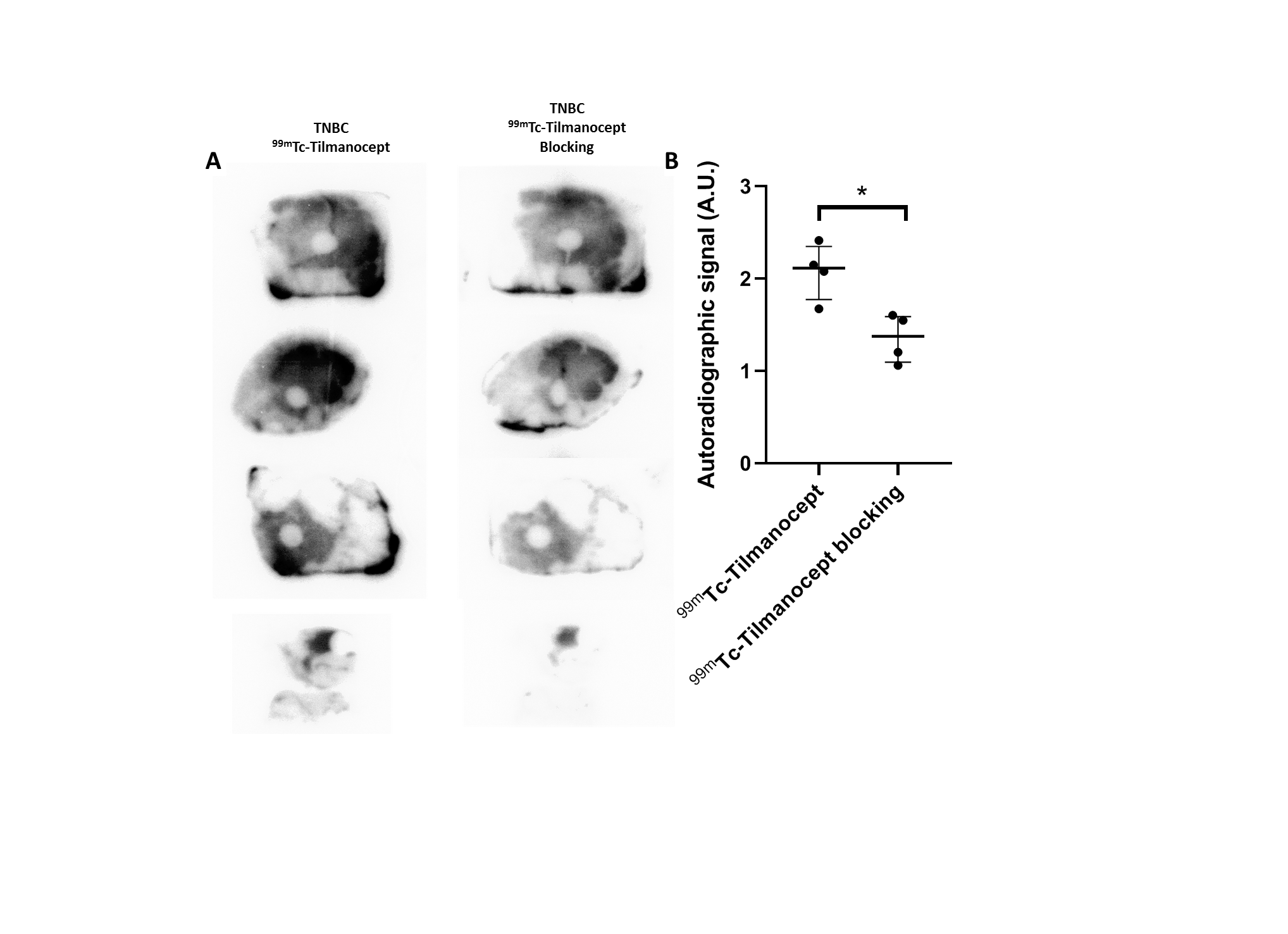

Supplement: Supplementary file 1 [file cells-10-03393-s001.zip › Figure S2.tif]

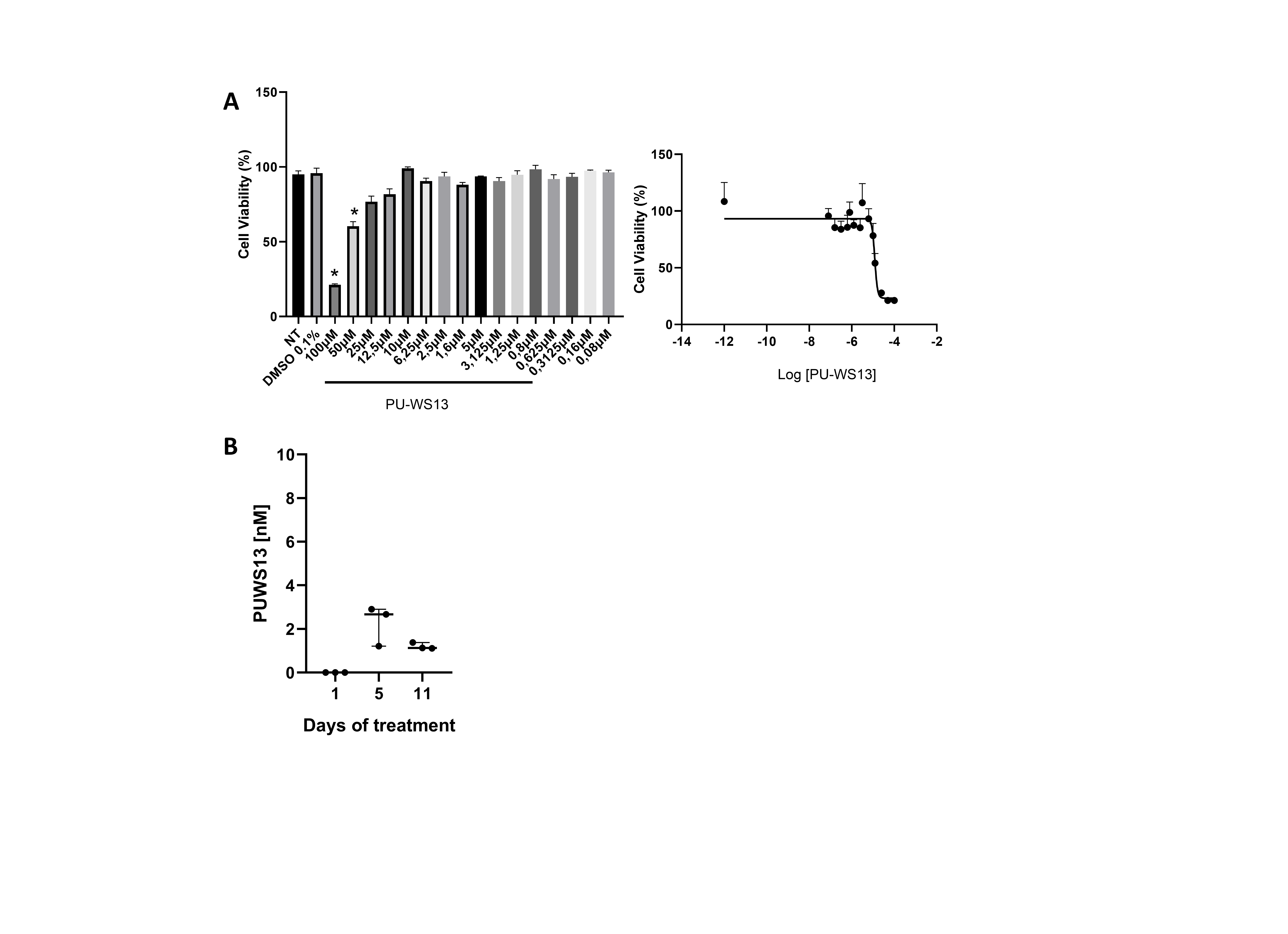

Supplement: Supplementary file 1 [file cells-10-03393-s001.zip › Figure S3.TIF]
